# How are generalist doctors made aware, on an ongoing basis, of the key new and updated clinical guidelines which are relevant to their practice? A systematic review

**DOI:** 10.1016/j.clinme.2025.100518

**Published:** 2025-09-29

**Authors:** Clare E Leong, Leonie Kallis, Isla L Kuhn, Graham P Martin, Zoë Fritz

**Affiliations:** a THIS Institute (The Healthcare Improvement Studies Institute), University of Cambridge, Strangeways Research Laboratory, 2 Worts’ Causeway, Cambridge CB1 8RN, UK; b School of Clinical Medicine, University of Cambridge, Box 111, Cambridge Biomedical Campus, Cambridge, CB2 0SP, UK

## Abstract

**Introduction:**

Clinical guidelines are an essential component of evidence-based medicine, and doctors have a responsibility to stay up-to-date. However, doctors’ lack of awareness of publication of new guidelines may limit adherence and impact on patient care. This paper systematically reviews evidence on strategies used to make doctors aware of new/updated clinical guidelines.

**Methods:**

Electronic databases (Medline, Embase via Ovid) were searched 2004–2024 for papers examining such strategies. Experimental, observational and qualitative studies were included. Data was extracted and critically analysed. PRISMA guidelines were followed.

**Results:**

Fourteen relevant articles were identified; they were heterogeneous and the overall quality of evidence was poor. Two multifaceted interventions resulted in better adherence to some aspects of guidelines; some others showed improvements in self-reported awareness-related outcomes.

**Conclusion:**

The evidence in this area is currently insufficient to recommend any strategy. Future research should focus on evaluation of interventions using appropriate study designs and objective outcome measures.

## Highlights

• Awareness of new/updated clinical guidelines is an essential and rate-limiting step in the provision of up-to-date care by doctors, but the literature in this area is sparse. There are very few published interventions addressing the problem, and the ethical considerations have not been explored.
• Individual clinicians, institutions, professional bodies and guideline producers share responsibility for clinicians keeping up-to-date, but the delineation of this responsibility is unclear.
• There is currently no clear evidence for an effective strategy to disseminate all relevant new/updated guidelines to practising clinicians. Developing a robust approach requires further carefully designed research investigating the problem and evaluating potential solutions.

## Introduction

Clinical guidelines are an essential component of evidence-based medicine: they provide accurate, trustworthy information to clinicians about how to treat their patients, based on the latest evidence and expert consensus.^1^ It is not feasible for individual clinicians to keep up with the rapidly proliferating primary literature by themselves, even within a small specialty or clinical area.^2^ Evidence-based guidelines summarise and translate the relevant evidence into actionable recommendations that can be accessed by clinicians to optimise their practice.^3^

New clinical guidelines are created and updated frequently (in the UK, the National Institute for Health and Care Excellence, NICE, published 48 new guidelines in 2023, and updated a further 28),^4^ but there is currently no widespread systematic means of informing clinicians that they have been published. Uptake of new guidance can be slow and incomplete, resulting in suboptimal care where the new guidance is not applied.^5^

**Fig. 1:**
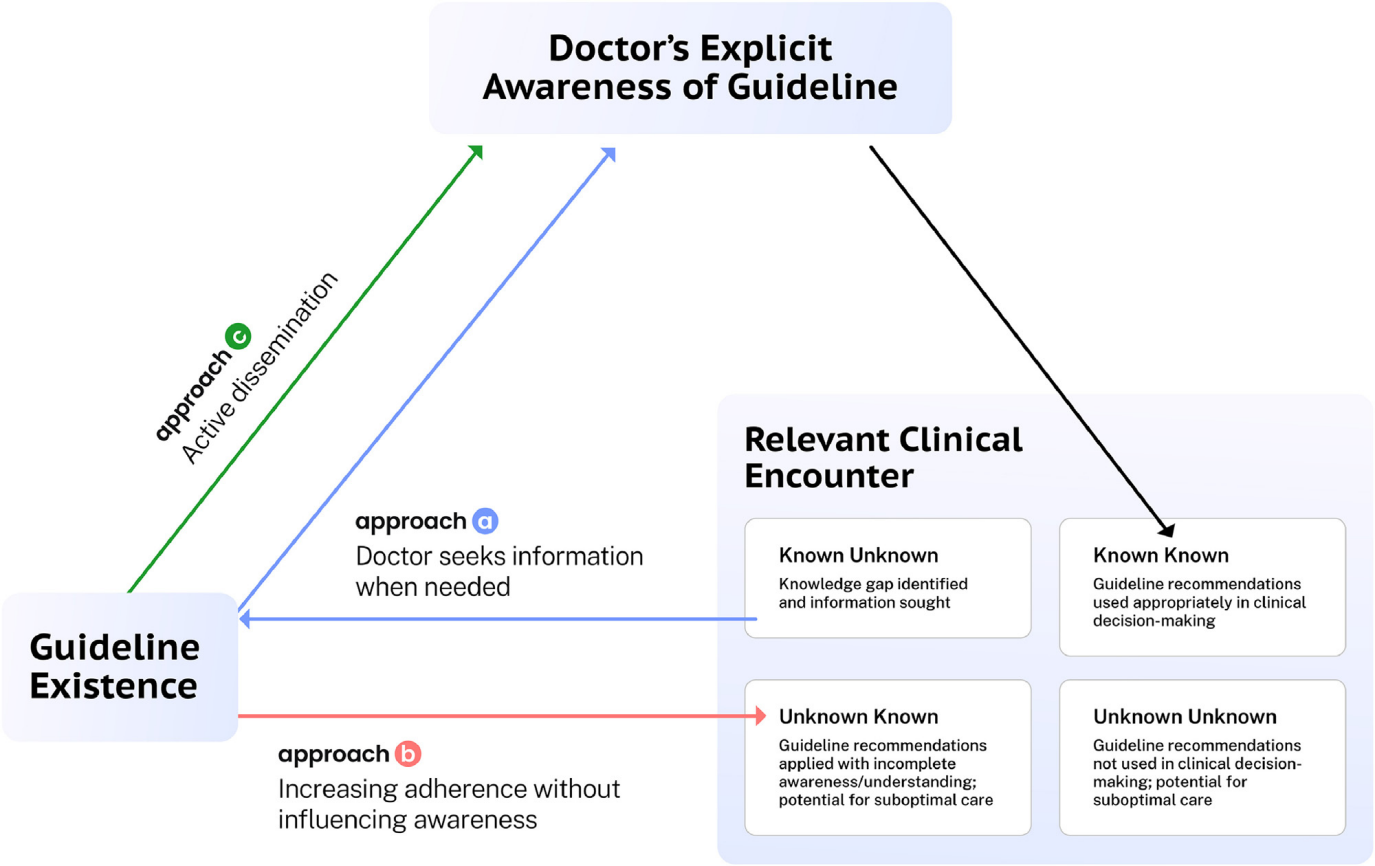
Approaches to improving clinicians’ uptake and use of guidelines.

Clinicians’ awareness of guidelines is a key and potentially rate-limiting early step in the process of guideline uptake and use. It has been presented as the first stage in a four-stage process: awareness; agreement; adoption; adherence.^6^ Improving the whole process might be approached in one of three ways (Fig. 1): a) making the guidelines readily available and relying on clinicians to seek out the guidelines when relevant to their practice; b) bypassing the awareness stage altogether and seeking to achieve adherence in other ways; or c) actively disseminating new guidelines to clinicians.

Approach a) underpins the traditional evidence-based model, which begins with the asking of a clinical question;^7^ numerous point-of-care resources exist to facilitate the retrieval of information (including guidelines) for this purpose.^8^ Although these have the potential to increase clinicians’ awareness of relevant information, ethnographic research indicates that doctors rarely access guidelines in their practice.^9^^,^^10^ It seems logical, furthermore, that if doctors do seek information to answer a clinical question, this would only be in relation to an identified knowledge gap (‘known unknown’).^11^ If a doctor is not aware of a new guideline that has been published (‘unknown unknown’), they would have no reason to access information on the subject, instead relying on an existing ‘mindline’.

Approach b) includes mechanisms such as clinical decision support systems^12^ (guideline recommendations incorporated into electronic health systems, to ‘nudge’, remind or restrict clinicians to follow recommendations) and audit and feedback,^13^ which may have an effect on adherence without increasing explicit awareness.^6^ However, these approaches are not in keeping with the fundamental principles of evidence-based medicine.^14^ Adherence without awareness does not allow for ‘*the conscientious, explicit, and judicious use of current best evidence in making decisions about the care of individual patients*’^14^ or for the integration of a clinician’s individual expertise with the evidence (see Fig. 1). As Pathman *et al* have illustrated,^6^ in order to practise evidence-based medicine effectively, clinicians must be explicitly aware of the best available evidence, agree with it, adopt it and adhere to it – bypassing the awareness stage will not further this goal.

Most studies relating to approach c) discuss activities to promote the dissemination and implementation of *individual* guidelines.^15^, ^16^, ^17^, ^18^ To provide the highest-quality care, doctors need to be aware, on an ongoing basis, of all new/updated clinical guidelines that are relevant to their practice, as soon as possible after their publication. Generalist doctors in particular must stay abreast of current guidelines across a wide range of specialties. Targeted work to increase awareness of individual guidelines in isolation may contribute to this goal, but is unlikely to address the problem as a whole. It may even distort focus towards higher-profile areas, at the expense of guidelines that do not benefit from this emphasis.

An additional unresolved issue is where responsibility lies for maintaining clinicians’ knowledge of the latest evidence and guidelines. The General Medical Council (GMC) mandates that doctors ‘propose, provide or prescribe effective treatment based on the best available evidence’ and ‘must make sure that the information [they] give patients is clear, accurate and up-to-date, and based on the best available evidence’.^19^ While the GMC emphasises the obligation of individual doctors, it also notes the responsibility of institutions that employ them and of national bodies.^20^ Several authors have suggested strategies for individual doctors to undertake themselves,^21^^,^^22^ but there is little evidence for their effectiveness. Relying on individuals to set up their own solutions will not address the problem at scale, and may contribute to burnout. While individual doctors have a professional responsibility to stay up-to-date, institutions and professional bodies may have a responsibility to make this easy (or at least easier).

This review therefore aims to:

- describe the strategies used to make generalist doctors aware, on an ongoing basis, of the key new or updated clinical guidelines that are relevant to their practice
- assess the effectiveness of these strategies.

Secondarily, it aims to:

- identify who is responsible for ensuring that clinicians stayed up-to-date in each strategy, and whether any ethical concerns are considered by the authors of the strategies.

## Methods

The systematic review protocol was prepared using the Preferred Reporting Items for Systematic Reviews and Meta-Analyses (PRISMA) guideline^23^ and registered in the PROSPERO database (registration number 536843).^24^ The protocol is available at https://www.crd.york.ac.uk/PROSPERO/view/CRD42024536843. Table 1 shows the inclusion and exclusion criteria.

**Table 1:** Inclusion and exclusion criteria.

Inclusion criteria	Exclusion criteria
Language: English Date of publication: 2004–2024 Participants/population:- Generalist medical doctors working in any setting Intervention(s):- Strategies for relaying clinical guidelines relating to any aspect of generalist medical practice (may include general practice, emergency medicine, acute medicine, general medicine, general surgery, general paediatrics) - Strategies must relate to new guidelines and/or include potential for ongoing/longitudinal communication of new guidelines as they are published/updated	Article type: Editorials, letters, commentaries/comments and reviews Participants/population:- Non-doctors (eg nurses, allied health professionals, students, patients/public, policy-makers) - Specialist doctors (where the guidelines concerned are relevant only to specialist and not to generalist practice) Interventions: - Dissemination of material other than guidelines (eg primary literature) - Strategies focused on a different aspect of guideline implementation rather than increasing awareness (eg examining awareness/adherence/implementation of clinical practice guidelines only, including electronic support systems, with no consideration of *how* this is achieved, or of doctors’ awareness) - Information-seeking methods or strategies to stay up-to-date undertaken by individual doctors for themselves - One-off rather than ongoing programmes (ie single guideline, or small number of old guidelines)

Database searches of MEDLINE and Embase via the Ovid interface were conducted on 12 April 2024. The search strategy combined free-text and subject headings for adoption or awareness (34 terms) with clinical practice or treatment guidelines (eight terms), together with terms for dissemination/awareness (seven terms), combined with free-text and subject headings for doctors/generalists (25 terms). The full strategy can be found in the supplementary materials (Multimedia Component 1).

The reference lists of all relevant reviews identified by the search strategy and of all included papers were checked against the inclusion/exclusion criteria to identify any additional studies not found in the initial search (reference mining).

Abstracts were screened blindly and independently by two authors (CL and LK) within Rayyan (Qatar Foundation, Qatar). Disagreements were resolved through discussion with a third reviewer (ZF). After initial screening, three authors (CL, LK and ZF) reviewed full articles using predefined inclusion and exclusion criteria; disagreements were resolved through discussion with a fourth reviewer (GM). Data extraction using a standardised form (Multimedia Component 2) was performed by two reviewers (CL and LK) independently and compared. Data were extracted for any outcomes relating to awareness, agreement, adoption and adherence to clinical guidelines among doctors, with all available results for each outcome included. Risk of bias for each study was assessed by one reviewer (CL or ZF), using the Cochrane Risk of Bias Assessment tools where appropriate for the study – RoB 2 for randomised trials and ROBINS I for non-randomised studies – and the JBI critical appraisal tool for qualitative studies.^25^, ^26^, ^27^

We anticipated that heterogeneity of the included studies in terms of both design and outcome would limit the quantitative syntheses possible. We planned to group studies according to the type of intervention and to identify common themes in a narrative synthesis, synthesising data where possible in studies with sufficiently similar interventions and outcomes.

## Results

**Fig. 2:**
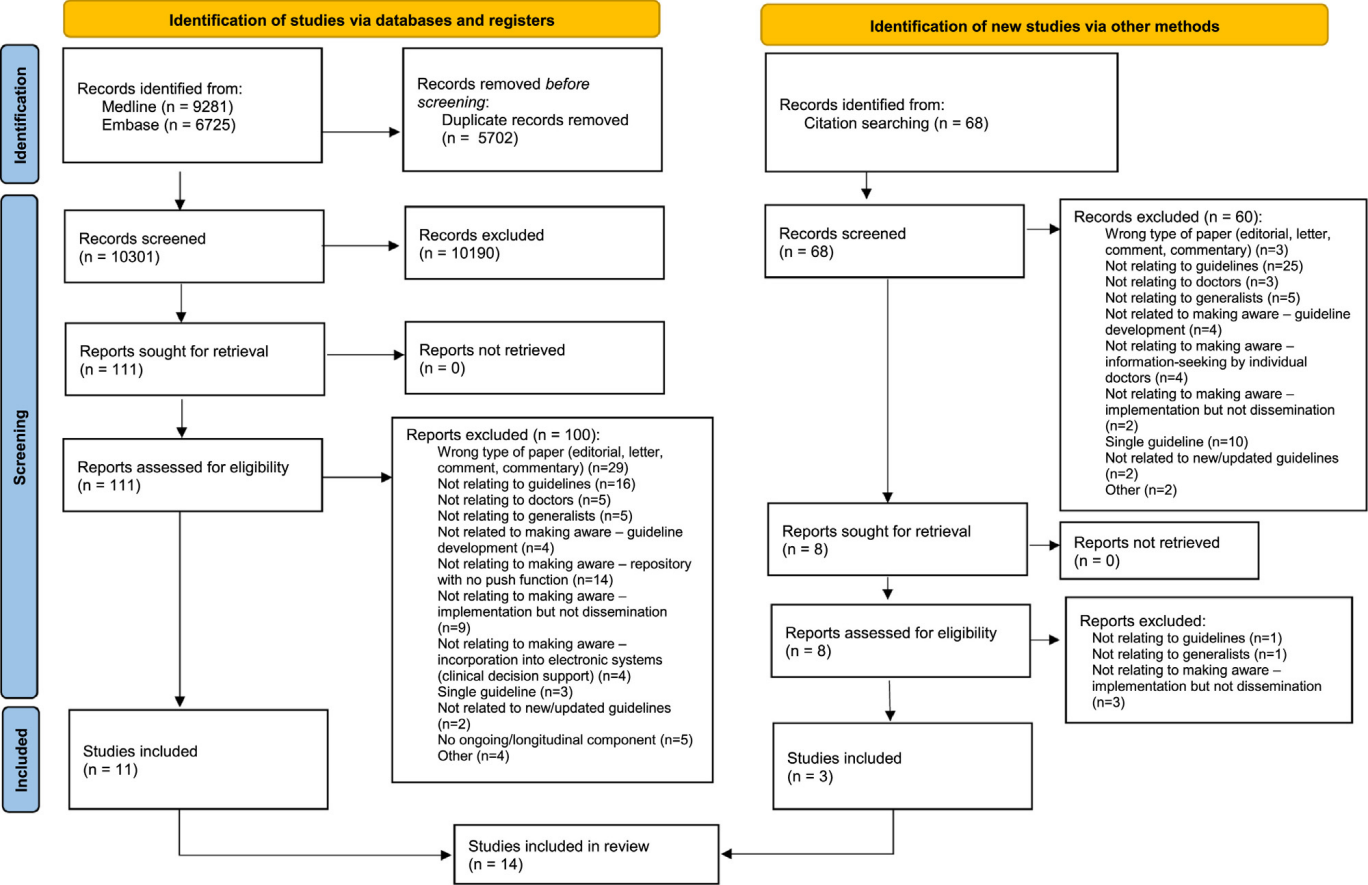
PRISMA flow diagram including reasons for exclusion.

The search yielded 10,301 papers, and a further 68 were found through reference mining; 119 of these were assessed more thoroughly for eligibility and 14 papers met inclusion criteria. See Fig. 2 for reasons for exclusion and PRISMA flow diagram. Details of publication, participants and guideline topics covered by the papers can be found in Table 2.

**Table 2:** Key characteristics of included studies.

Details of publication, participants and guideline					
References	Publication year	Location	Participants	Number of sites/participants	Guidelines
Amer *et al*^35^	2019	Saudi Arabia	Teaching hospital staff	One hospital	Multiple (various specialties)
Echlin *et al*^31^	2004	Canada	Family medicine residents	Two sites; 34 participants	Hypertension, hypercholesterolaemia
Etxeberria *et al*^34^	2018	Spain	Primary care doctors	43 primary care units (459 doctors)	Lipids, hypertension, type 2 diabetes
Fahim *et al*^30^	2023	Canada	Primary care doctors	1,284 participants (survey); 183 (interviews)	Preventive health and screening
Farrell *et al*^38^	2022	Designed in Canada; international audience	Doctors, pharmacists, nurses, other non-doctors	161 physician respondents (261 non-doctor respondents)	Deprescribing
Fleuren *et al*^37^	2015	Netherlands	Doctors, nurses, doctor’s assistants (preventive child healthcare organisations)	82–417 respondents	Preventive child health care
Fretheim *et al*^33^	2006	Norway	Primary care doctors	139 practices (501 physicians)	Hypertension, hypercholesterolaemia
Johnson *et al*^40^	2004	USA	Faculty members from school of medicine, resident doctors, practice nurses, human resources specialist	16 participants (11 doctors, five non-doctors)	Topics not specified
Kumar and Bhardwaj^36^	2019	India	Doctors working at state government-run medical colleges	335 participants	Tuberculosis
Niquille *et al*^28^	2010	Switzerland	Primary care doctors	Six groups	Prescribing
Pinto *et al*^29^	2018	Portugal	Primary care doctors	38 practices with 239 physicians (120 intervention, 119 control)	Prescribing
Thies *et al*^41^	2021	USA	Staff from community health centres, hospital systems, government agencies, universities and community-based organisations	454 respondents	COVID-19
Tso^39^	2022	China	Doctors, technicians, nurses, pharmacists, public health administrators	58 participants (39 doctors) across 24 healthcare settings	Not specified
Van Harrison *et al*^32^	2006	USA	Primary care doctors	Three hospitals and 14 ambulatory care centres with 228 physicians	24 common conditions in primary care

Table 3 provides a summary of findings, including interventions, scope of guidelines being implemented, study design, effectiveness of interventions in increasing awareness, adherence or adoption, and risk of bias assessments. Here we describe the contexts in which the studies occurred, the variety of study designs used, and the scope of guidelines being disseminated, before providing a narrative description of the nature and impact of the strategies used.

**Table 3:** Details of interventions and study designs presented in included sources.

Details of intervention, guideline scope, sesign, outcomes and risk of bias							
References	Intervention	Guideline scope	Design	Comparator	Outcomes	Results	Risk of bias – tool used and bias assessment
*Multifaceted interventions*							
Fretheim *et al*^33^	Multifaceted, including educational outreach visit, direct distribution, audit and feedback, computerised reminders	Defined number, preselected	Cluster-randomised trial	Control group (usual guideline implementation via passive dissemination)	Guideline **a** **dherence** (prescribing and patient care data)	**Statistically significant improvement in one primary outcome (thiazide prescribing) in intervention group compared to control group;** no other significant differences in primary or secondary outcomes between groups	Cochrane RoB 2 Tool: ‘Some concerns’ for most outcomes due to lack of blinding and insufficient explanation for change in analysis plan. ‘High risk’ for cardiovascular risk assessment due to unreliable measurement of outcome.
Etxeberria *et al*^34^	Multifaceted, including presentation by individuals involved in guideline development, workshops, and access to a website with tools to assist application	Defined number, preselected	Cluster-randomised trial	Control group (usual guideline implementation via email, publishing on website and presentation at meetings)	Guideline **a** **dherence** (patient care data, clinical outcomes)	**Statistically significant improvement in one primary outcome (cardiovascular risk assessment in dyslipidaemia) in intervention group compared to control group;** no other significant differences in primary outcomes between groups	Cochrane RoB (Risk of Bias) 2 Tool: ‘Some concerns’ – insufficient information presented to confirm low risk of bias across multiple domains.
Fleuren *et al*^37^	Multifaceted, including dissemination of guidelines to all professionals (route not specified), training on guideline content offered to all professionals, evaluation of guideline use after publication, employment of implementation coordinators in each organisation, and a national help desk relating to the guidelines.	All from single organisation in relevant time period	Cross-sectional evaluation	Pre-intervention data (limited)	Self-reported **awareness**, guideline **a** **dherence** (‘level of use’)	**Change over time (statistically significant improvement) reported for only two of multiple guidelines discussed**.	Cochrane ROBINS-I tool: Critical risk of bias due to confounding being inherently non-controllable.
Fahim *et al*^30^	Multifaceted, including publication, direct distribution (eg at conferences), advertisements in media and social media, and webinars	All from single organisation in relevant time period	Cross-sectional annual evaluation	None	Self-reported **awareness**	Variable awareness of different guidelines	Cochrane ROBINS-I tool: Serious risk of bias in measurement of outcomes (subjective and assessors aware of intervention).
*Individuals meeting doctors*							
Pinto *et al*^29^	Individual meetings with doctors led by ‘detailers’, defined as ‘healthcare professionals trained in communication skills and educational outreach’	Defined number, preselected	Cluster-randomised trial	Control group (usual guideline implementation via passive dissemination)	Guideline **a** **dherence** (prescribing data)	No significant difference between groups	Cochrane RoB 2 Tool: ‘Some concerns’ due to lack of blinding.
Niquille *et al*^28^	Individuals meeting doctors (pharmacists)	Defined number, preselected	Time series with control group	Control group (no intervention)	Guideline **a** **dherence** (prescribing data)	Intervention group more adherent than control group. Statistical significance not reported.	Cochrane ROBINS-I tool: Serious risk of bias due to confounding.
*Regular updates*							
Thies *et al*^41^	Regular updates (weekly webinars)	New/updated, from multiple sources on one clinical topic	Post- intervention evaluation	None	Self-reported **awareness, agreement** and **adoption** (change in knowledge, intention to change practice)	Majority reported knowledge gain and/or intention to change practice	Cochrane ROBINS-I tool: Serious risk of bias in measurement of outcomes (subjective and assessors aware of intervention).
Johnson *et al*^40^	Alert system with headlines sent to personal digital assistants and full details available by email	New/updated, from National Guidelines Clearinghouse (also alerts for non-guideline resources, such as primary literature)	Post-intervention evaluation	None	Usage of system (headline requests); self-reported **awareness** (usefulness for keeping up to date)	50% of respondents found the system somewhat to extremely useful for keeping up to date. 50% reported learning about new medical developments that they would not otherwise have learned about.	Cochrane ROBINS-I tool: Serious risk of bias in measurement of outcomes (subjective and assessors aware of intervention).
Kumar and Bhardwaj^36^	Monthly system of text messages to convey key points from the guidelines	All from single organisation in relevant time period	Description of intervention	None	None specified	No results presented	Unable to assess.
*Development of local guidelines*							
Van Harrison *et al*^32^	Development of local guidelines via adaptation, with multifaceted dissemination	Defined number, preselected	Time series	Pre-intervention data	Guideline **adherence** (patient care data)	Improvements over time; statistical significance not reported	Cochrane ROBINS-I tool: Serious risk of bias due to confounding.
Amer *et al*^35^	Development of local guidelines via adaptation, with multifaceted dissemination	Defined number, preselected	Description of intervention	None	None specified	No results presented	Unable to assess.
*Other interventions*							
Echlin *et al*^31^	Distribution of hard copies	Defined number, preselected	Pre- and post-intervention evaluation	Pre-intervention data	Self-reported (familiarity) and objective (knowledge assessment) **awareness** and **agreement**	Significant improvement in post-intervention self-reported familiarity; no difference in objectively assessed knowledge	Cochrane ROBINS-I (Risk of Bias in non-randomised studies of interventions) tool: Critical risk of bias due to confounding being inherently non-controllable.
Farrell *et al*^38^	Creation of supporting content (YouTube videos)	Defined number, preselected	Post-intervention evaluation	None	Self-reported (change in knowledge) **a** **wareness** and **agreement**	Minority reported gaining knowledge	Cochrane ROBINS-I tool: Serious risk of bias in measurement of outcomes (subjective and assessors aware of intervention).
Tso^39^	Sharing of guidelines via social media (WeChat)	New/updated, from multiple sources	Qualitative	None	Not prespecified	Themes emerged of intervention facilitating access to and application of guidelines	JBI (Joanna Briggs Institute) critical appraisal tool for qualitative studies: Overall appraisal was to include, although limitations were that the researcher was not explicitly located culturally or theoretically within the manuscript, and the influence of the researcher on the research, and vice versa, was not addressed

### Context

The studies were geographically diverse. Seven studies concerned primary care / family medicine doctors,^28^, ^29^, ^30^, ^31^, ^32^, ^33^, ^34^ two targeted hospital doctors,^35^^,^^36^ one dealt with healthcare professionals in child health organisations,^37^ and four were aimed at a broader medical audience.^38^, ^39^, ^40^, ^41^

The clinical guidelines covered were similarly varied, relating to: prescribing^28^^,^^29^ or deprescribing;^38^ preventive care generally^30^ or for children specifically;^37^ COVID-19;^41^ cardiovascular and related conditions;^31^^,^^33^^,^^34^ tuberculosis;^36^ and multiple topics in primary care^32^ or multiple specialties.^35^ Two studies^39^^,^^40^ did not specify the disseminated guidelines.

### Study design and risk of bias

Three of the studies were cluster-randomised trials (CRTs)^29^^,^^33^^,^^34^; all three had ‘some concerns’ for risk of bias. Eight used quasi-experimental designs (eg cohort study, before and after time series), although only one of these had a contemporaneous control group,^28^ and all had a serious or critical risk of bias. Two described interventions but did not evaluate their effectiveness.^35^^,^^36^ The final study was a qualitative investigation into a process through which doctors shared guidelines themselves.^39^

Of the 11 studies that performed some quantitative evaluation of effectiveness, six measured outcomes self-reported by doctors, including awareness of guidelines, change in knowledge and intention to change their practice.^30^^,^^31^^,^^37^^,^^38^^,^^41^ Johnson *et al* also measured usage of the dissemination system (via technical data).^40^ One study used objective assessments of doctors’ knowledge of guideline content.^31^ Six measured guideline adherence via data collected in the process of patient care.^28^^,^^29^^,^^32^, ^33^, ^34^ Etxeberria *et al* also measured clinical outcomes.^34^

### Guideline scope

Three articles described interventions by guideline-generating organisations for all new guidelines that they published over a defined period.^30^^,^^36^^,^^37^ Three other studies discussed strategies to make doctors aware of new/updated guidelines from multiple sources on a regular basis.^39^, ^40^, ^41^

The remaining eight articles described interventions to disseminate a fixed number of preselected guidelines.^28^^,^^29^^,^^31^, ^32^, ^33^, ^34^, ^35^^,^^38^ All the interventions related to recently published or updated guidelines and included at least the potential for continued updating on an ongoing basis, but none of these studies discussed or evaluated the incorporation of newly published/updated guidelines in real time. Two studies focused on the adaptation of national/international guidelines to develop local guidelines, in conjunction with additional dissemination and implementation activities.^32^^,^^35^

### Nature and impact of intervention

Four studies used multifaceted interventions (details in Table 3) to increase guideline use. Fretheim *et al* and Etxeberria *et al* evaluated prescribing and patient care data to assess guideline adherence in CRTs,^33^^,^^34^ with Etxeberria *et al* also including clinical endpoints as secondary outcomes.^34^ Each found a statistically significant improvement in one primary outcome in the intervention group compared to the control group (completion of cardiovascular risk assessments^34^ and prescribing of a thiazide as the first-line agent for newly diagnosed hypertension,^33^ with no other significant differences between groups in primary outcomes. Etxeberria *et al* found additional statistically significant differences in favour of the intervention group relating to several secondary outcomes evaluating guideline adherence, but no differences in clinical outcomes between groups.^34^ Fleuren *et al* and Fahim *et al* each provided cross-sectional evaluations of self-reported awareness of guidelines.^30^^,^^37^ Fahim *et al* did not provide any comparators or assessment of the effect of the intervention.^30^ Fleuren *et al* measured awareness at multiple timepoints for three guidelines (but at only one timepoint for other included guidelines): one demonstrated statistically significant improvement in awareness, one a statistically significant worsening, and one no change.^37^

Of the two studies that involved individuals meeting doctors to discuss and promote guideline use, Pinto *et al*’s was more robust, using a CRT. They found no significant differences in guideline adherence between intervention and control groups.^29^ Niquille *et al* reported greater adherence among the intervention group than the control group, but did not provide data on statistical significance.^28^

Three studies described the provision of regular updates to doctors through various means. The majority of respondents in Thies *et al*’s study using weekly webinars reported knowledge gain and/or an intention to change their practice following the intervention.^41^ In Johnson *et al*’s study using a digital alerting system, eight of 16 respondents found the system somewhat to extremely useful for keeping up-to-date, and eight of 16 reported learning about new medical developments that they would not otherwise have heard about.^40^ Kumar *et al*’s study of the use of text messages to provide regular updates was descriptive only, and contained no evaluation of impact.^36^

Echlin *et al* assessed direct distribution of physical copies of guidelines to clinicians, and found no change in objectively assessed knowledge among participants, despite an improvement in self-reported familiarity with the guidelines.^31^ Farrell *et al* evaluated the creation of YouTube videos to support guidelines. The study assessed change in knowledge via self-report only, with only a minority of participants indicating that their knowledge had improved.^38^ Tso’s qualitative study of sharing of guidelines via social media (WeChat) suggested that it led to improved access to and application of guidelines.^39^

Of the two studies describing the development of local guidelines via adaptation of existing national or international guidelines, Van Harrison *et al* reported improvements in guideline adherence over time, but did not comment on statistical significance.^32^ Amer *et al* presented their intervention alone, with no evaluation of its impact.^35^

### Ethical considerations

Two papers briefly considered the question of where responsibility lies for making doctors aware of guidelines: Kumar and Bhardwaj commented that ‘public health has a mandate’ to share updated guidelines,^36^ while Fleuren *et al* mentioned that some stakeholders had initially believed that ‘the professionals themselves were fully responsible for implementation’ of guidelines.^37^ No other ethical issues were discussed in any of the articles.

## Discussion

If doctors are to provide care to their patients that adheres to the latest guidelines, they must have some means of becoming aware of the publication of new/updated guidelines from various sources on an ongoing basis. Our review revealed very few published interventions intended to achieve this objective, with even fewer being robustly evaluated to demonstrate a difference in knowledge or practice, and no clear evidence of effectiveness for any intervention. None of the studies explored the ethical question of who holds responsibility for ensuring doctors stay up-to-date.

Only three studies covered new and updated guidelines from multiple sources. One of these investigated a pre-existing process by which doctors shared guidelines on social media,^39^ rather than testing a new intervention. Another study, involving weekly webinars,^41^ took place in the unique context of the COVID-19 pandemic and focused specifically on knowledge relating to COVID-19; its results may not be generalisable to the way doctors learn in more ‘normal’ times and across the whole scope of practice, although they may help with pandemic preparedness.^42^ The third study used a digital alert-based system to update users about new guidelines and other resources:^40^ while the intervention may show potential in addressing the problem, its evaluation was very limited in terms of study design, measured outcomes and number of participants.

The other included articles were all limited either to guidelines produced by a single organisation, or to a defined number of preselected guidelines. While some of the interventions may be adaptable to incorporate guidelines from multiple sources and/or new/updated guidelines as they are published, this has not yet been evaluated. The low quality of the majority of included studies also severely limits the extent to which any conclusions can be drawn regarding the effectiveness of the specified interventions.

This systematic review is robust in that it was conducted across two databases with study identification and data extraction being carried out by two blinded reviewers according to stringent and reproducible criteria. Limitations include the exclusion of the grey literature, and the heterogenous nature of the studies restricting data comparison.

Only two interventions centred around active dissemination of guidelines to individuals via mobile phones (text messages) or digital alerts,^36^^,^^40^ with none focusing on email. We chose to search papers from 2004 because this was the year that nhs.net email addresses were instituted in the UK: from this time, all doctors in the NHS could be contacted and receive updates via email, and yet no studies were found which tested this approach. There are systems in place for emailing guideline updates (for example, in the UK, clinicians can subscribe to NICE alerts),^43^ but we did not identify any literature relating to their impact or effectiveness. Email and app-based alerts have been demonstrated to have some effectiveness in increasing clinicians’ awareness of the primary literature, but this has not been studied with regard to guidelines.^44^^,^^45^ Evaluating interventions in the context of clinical guidelines for generalist doctors would represent a logical avenue for future research. Doctors are perhaps unusual in the *amount* of updated information they need to keep abreast of, but other professions outside healthcare have made inroads: the European Food Safety Authority, for example, has published recommendations on how to share updates, including defining responsibilities and timelines for communications.^46^

The absence of any discussion of responsibility for ensuring that doctors are up-to-date with guidelines (or any other ethical considerations relating to this issue) in the existing literature is noteworthy. Lack of clarity about who holds responsibility for ensuring doctors stay up-to-date may contribute to burnout^47^, ^48^, ^49^ and moral injury,^50^ for example if doctors feel that they cannot deliver the best for their patients because they are unable to stay up-to-date. These issues are particularly pressing in the context of a stretched health service, where resident doctors in particular are less supported than their predecessors for various reasons, including a loss of the ‘firm’ structure and the omnipresence of work in a digital age.^51^ While employers and individual doctors have a GMC-mandated responsibility to provide up-to-date care,^19^ the moral responsibility may lie with the professional bodies. Organisations such as UK royal colleges and their equivalents should consider their potential role in systematically improving the awareness of the latest guidelines among doctors as part of their commitment to continuing professional development, in addition to the conferences and journal they provide. A system-wide approach to disseminating guidelines on a national scale would be efficient and equitable and remove some of the burden of responsibility from individual doctors.

While it is important to think about *who* should have responsibility for disseminating guidelines, further work is clearly needed to determine *effective* strategies for doing so. The quality of the studies we identified is not strong enough for us to inform recommendations on which interventions are effective, and so significant further work needs to be done. Cross-sectional evaluations were common among the papers in this review; future studies must have comparators (whether pre-intervention data on the same population, or a control group) in order to determine the extent to which any impact from the intervention is causal. Considering which outcome measures are best for assessing the effectiveness of interventions is essential, alongside reducing the risk of bias. While there is some research on understanding users’ needs,^52^^,^^53^ further work on optimising guidelines and minimising alert fatigue needs to be done.

Although it is tempting to seek solutions that address the whole process of implementation (from the existence of guidelines to incorporation into patient care), this is a complex field; two decades of implementation research have not produced any straightforward answers,^54^ and confounding factors make evaluating the impact of the dissemination of guidelines difficult. *Awareness* of guidelines is an important rate-limiting step in the process of implementation, and our understanding of how to improve this is currently very limited.

## Conclusions

Future efforts to improve the dissemination of guidelines should be accompanied by robust evaluation of awareness and impacts on practice. A trustworthy system for distributing guidance in a way that is effective in keeping doctors up-to-date would be invaluable for doctors themselves, for the healthcare system and for patients.

## Funding

CL is undertaking a Wellcome-funded clinical PhD programme (grant number 317451/Z/24/Z; commenced December 2023).

ZF is funded in part by the 10.13039/100010269Wellcome Trust 208213/Z/17/Z and part-funded by the Health Foundation's grant to the 10.13039/501100000580University of Cambridge for The Healthcare Improvement Studies (THIS) Institute (RG88620).

GM is funded by the Health Foundation’s grant to the 10.13039/501100000580University of Cambridge for The Healthcare Improvement Studies (THIS) Institute (RG88620).

## CRediT authorship contribution statement

**Clare E Leong:** Writing – review & editing, Writing – original draft, Methodology, Investigation, Funding acquisition, Formal analysis, Conceptualization. **Leonie Kallis:** Writing – review & editing, Formal analysis. **Isla L Kuhn:** Writing – review & editing, Software, Methodology, Data curation. **Graham P Martin:** Writing – review & editing, Supervision, Formal analysis, Conceptualization. **Zoë Fritz:** Writing – review & editing, Supervision, Methodology, Formal analysis, Conceptualization.

## Declaration of competing interest

The authors declare the following financial interests/personal relationships which may be considered as potential competing interests:

Zoe Fritz reports financial support was provided by Wellcome Trust. Zoe Fritz reports financial support was provided by The Health Foundation. Graham Martin reports financial support was provided by The Health Foundation. Isla Kuhn reports financial support was provided by The Health Foundation. Clare Leong reports financial support was provided by Wellcome Trust. If there are other authors, they declare that they have no known competing financial interests or personal relationships that could have appeared to influence the work reported in this paper.

## Appendix Supplementary materials

Search strategy

Data extraction form

PRISMA checklist
