# Supplementary material for: How are generalist doctors made aware, on an ongoing basis, of the key new and updated clinical guidelines which are relevant to their practice? A systematic review
Source: Clin Med (Lond). 2025 Sep 29;25(6):100518. doi: 10.1016/j.clinme.2025.100518 (PMC12615289; doi:10.1016/j.clinme.2025.100518)
Supplement: Supplementary file 1 [file mmc1.docx]

| Medline via Ovid | 9,281 |  |
| --- | --- | --- |
| Embase via Ovid | 6,725 |  |
| Total | 16,006 |  |
| Total dedpulicated | 10,304 |  |

Ovid MEDLINE(R) and Epub Ahead of Print, In-Process, In-Data-Review & Other Non-Indexed Citations, Daily and Versions <1946 to April 12, 2024>

1 ((implement* or translat* or transfer* or communicat* or share* or sharing or disseminat* or mobilis* or mobiliz* or distribut* or promot* or advertis* or advertiz* or diffus* or alert* or highlight* or utilis* or utiliz* or uptake or aware* or adopt* or incorporat* or updat* or "find out" or "finding out" or "keep up" or "keeping up" or "up to date" or discover or "hear* about" or learn* or source* or familiar* or "knowledge broker*") adj2 (guideline* or guidance or clinical knowledge or medical knowledge)).ti,ab. 26144

2 ((implement* or translat* or transfer* or communicat* or share* or sharing or disseminat* or mobilis* or mobiliz* or distribut* or promot* or advertis* or advertiz* or diffus* or alert* or highlight* or utilis* or utiliz* or uptake or aware* or adopt* or incorporat* or updat* or "find out" or "finding out" or "keep up" or "keeping up" or "up to date" or discover or "hear* about" or learn* or source* or familiar* or "knowledge broker*") adj2 (clinical practice guideline* or clinical practice guidance or clinical guideline* or clinical guidance or treatment guideline* or treatment guidance or CPG or CPGs)).ti,ab. 6185

3 exp Practice Guidelines as Topic/ or Guideline Adherence/ 148991

4 Information Dissemination/ or Diffusion of Innovation/ or Implementation Science/ or Translational Science, Biomedical/ or Education, Medical, Continuing/ or Scholarly Communication/ or Interdisciplinary Communication/ 81850

5 3 and 4 3551

6 1 or 2 or 5 33603

7 (doctor or doctors or clinician* or generalist* or hospitalist* or physician* or medic* specialist* or internist* or healthcare worker* or health care worker* or healthcare personnel or health care personnel or healthcare professional* or health care professional* or health professional* or health worker* or "hospital setting*" or practitioner* or healthcare provider* or health care provider*).ti,ab. or Physicians/ or General Practitioners/ or Hospitalists/ or Family Physicians/ or Primary Care Physicians/ 1332578

8 6 and 7 9848

9 ((implement* or translat* or transfer* or communicat* or share* or sharing or disseminat* or mobilis* or mobiliz* or distribut* or promot* or advertis* or advertiz* or diffus* or alert* or highlight* or utilis* or utiliz* or uptake or aware* or adopt* or incorporat* or updat* or "find out" or "finding out" or "keep up" or "keeping up" or "up to date" or discover or "hear* about" or learn* or source* or familiar* or "knowledge broker*") and (clinical guideline* or clinical guidance or clinical knowledge or medical knowledge or clinical practice guideline* or clinical practice guidance or treatment guideline* or treatment guidance)).ti. 1886

10 8 or 9 11279

11 limit 10 to yr="2004 -Current" 9823

12 limit 11 to english language 9281

Embase <1974 to 2024 April 12>

1 ((implement* or translat* or transfer* or communicat* or share* or sharing or disseminat* or mobilis* or mobiliz* or distribut* or promot* or advertis* or advertiz* or diffus* or alert* or highlight* or utilis* or utiliz* or uptake or aware* or adopt* or incorporat* or updat* or "find out" or "finding out" or "keep up" or "keeping up" or "up to date" or discover or "hear* about" or learn* or source* or familiar* or "knowledge broker*") adj2 (guideline* or guidance or clinical knowledge or medical knowledge)).ti,ab. 39333

2 ((implement* or translat* or transfer* or communicat* or share* or sharing or disseminat* or mobilis* or mobiliz* or distribut* or promot* or advertis* or advertiz* or diffus* or alert* or highlight* or utilis* or utiliz* or uptake or aware* or adopt* or incorporat* or updat* or "find out" or "finding out" or "keep up" or "keeping up" or "up to date" or discover or "hear* about" or learn* or source* or familiar* or "knowledge broker*") adj2 (clinical practice guideline* or clinical practice guidance or clinical guideline* or clinical guidance or treatment guideline* or treatment guidance or CPG or CPGs)).ti,ab. 8713

3 exp *practice guideline/ 130099

4 exp *protocol compliance/ 5309

5 3 or 4 134135

6 exp *information dissemination/ 5975

7 exp *"diffusion of innovation"/ 74

8 exp *implementation science/ 2137

9 exp *translational science/ 643

10 exp *medical education/ or exp *continuing education/ 187105

11 exp *scholarly communication/ 117

12 exp *interdisciplinary communication/ 4306

13 6 or 7 or 8 or 9 or 10 or 11 or 12 199864

14 5 and 13 1474

15 1 or 2 or 14 46765

16 (doctor or doctors or clinician* or generalist* or hospitalist* or physician* or medic* specialist* or internist* or healthcare worker* or health care worker* or healthcare personnel or health care personnel or healthcare professional* or health care professional* or health professional* or health worker* or "hospital setting*" or practitioner* or healthcare provider* or health care provider*).ti,ab. 1775275

17 exp *physician/ 210805

18 exp *general practitioner/ 29086

19 exp *medical staff/ 12441

20 16 or 17 or 18 or 19 1890084

21 15 and 20 14037

22 ((implement* or translat* or transfer* or communicat* or share* or sharing or disseminat* or mobilis* or mobiliz* or distribut* or promot* or advertis* or advertiz* or diffus* or alert* or highlight* or utilis* or utiliz* or uptake or aware* or adopt* or incorporat* or updat* or "find out" or "finding out" or "keep up" or "keeping up" or "up to date" or discover or "hear* about" or learn* or source* or familiar* or "knowledge broker*") and (clinical guideline* or clinical guidance or clinical knowledge or medical knowledge or clinical practice guideline* or clinical practice guidance or treatment guideline* or treatment guidance)).ti. 2394

23 21 or 22 15840

24 limit 23 to yr="2004 -Current" 14388

25 limit 24 to english language 13725

26 limit 25 to embase 6725

Note: The initial record stated that a sample of the papers identified by the search would be screened for eligibility by two reviewers independently to check concordance, with the remainder screened by one reviewer only. Following registration and completion of the initial search but prior to the screening process, the registered record was amended to state that all of the studies would be screened independently by two reviewers for eligibility.
